# Supplementary material for: Will Households Invest in Safe Sanitation? Results from an Experimental Demand Trial in Nakuru, Kenya
Source: Int J Environ Res Public Health. 2021 Apr 22;18(9):4462. doi: 10.3390/ijerph18094462 (PMC8122837; doi:10.3390/ijerph18094462)
Supplement: Supplementary file 1 [file ijerph-18-04462-s001.zip › ijerph-1143245-supplementary.pdf]

Supplementary Materials

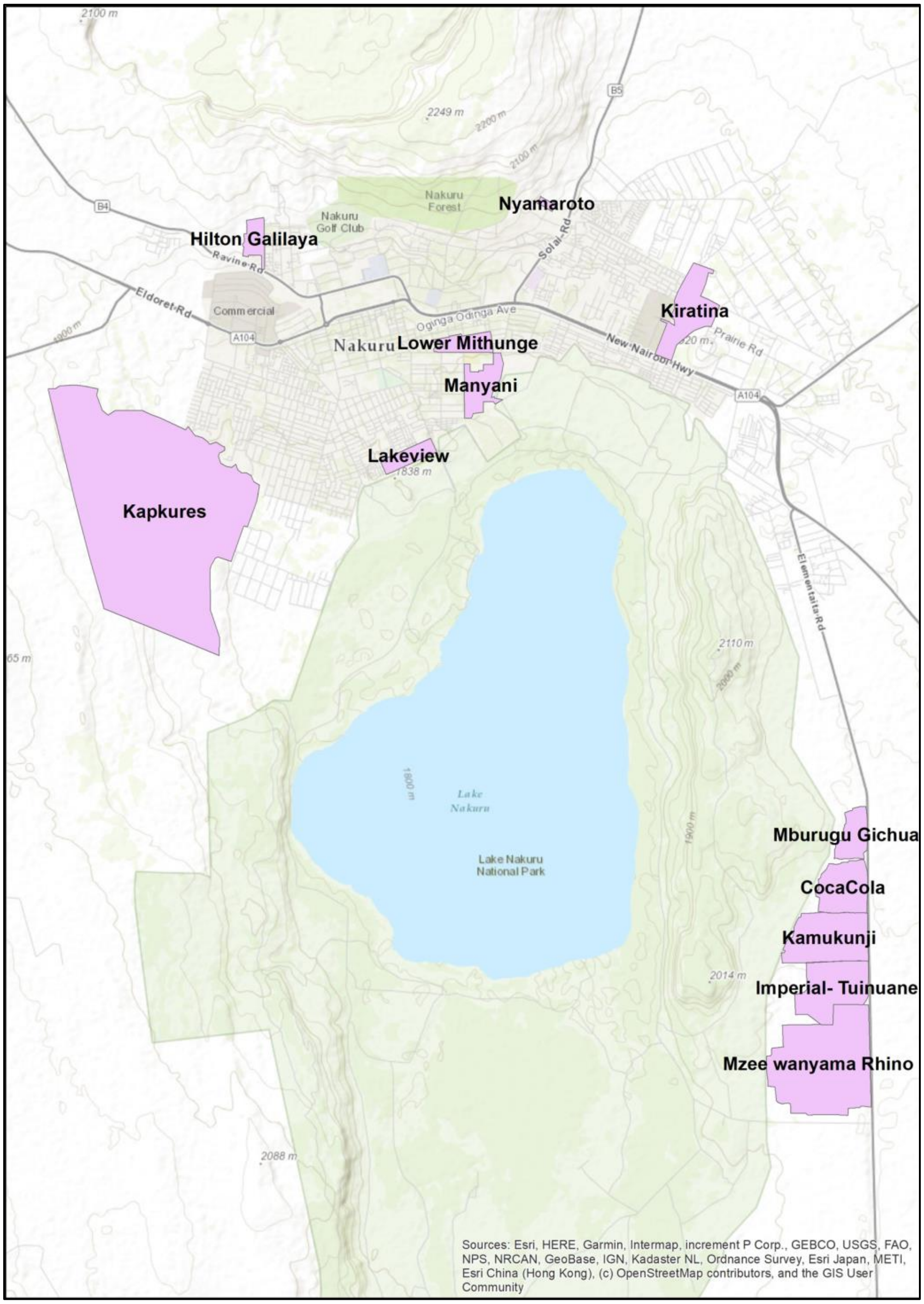

Figure S1. Study locations (12 total) in the low-income areas of Nakuru.

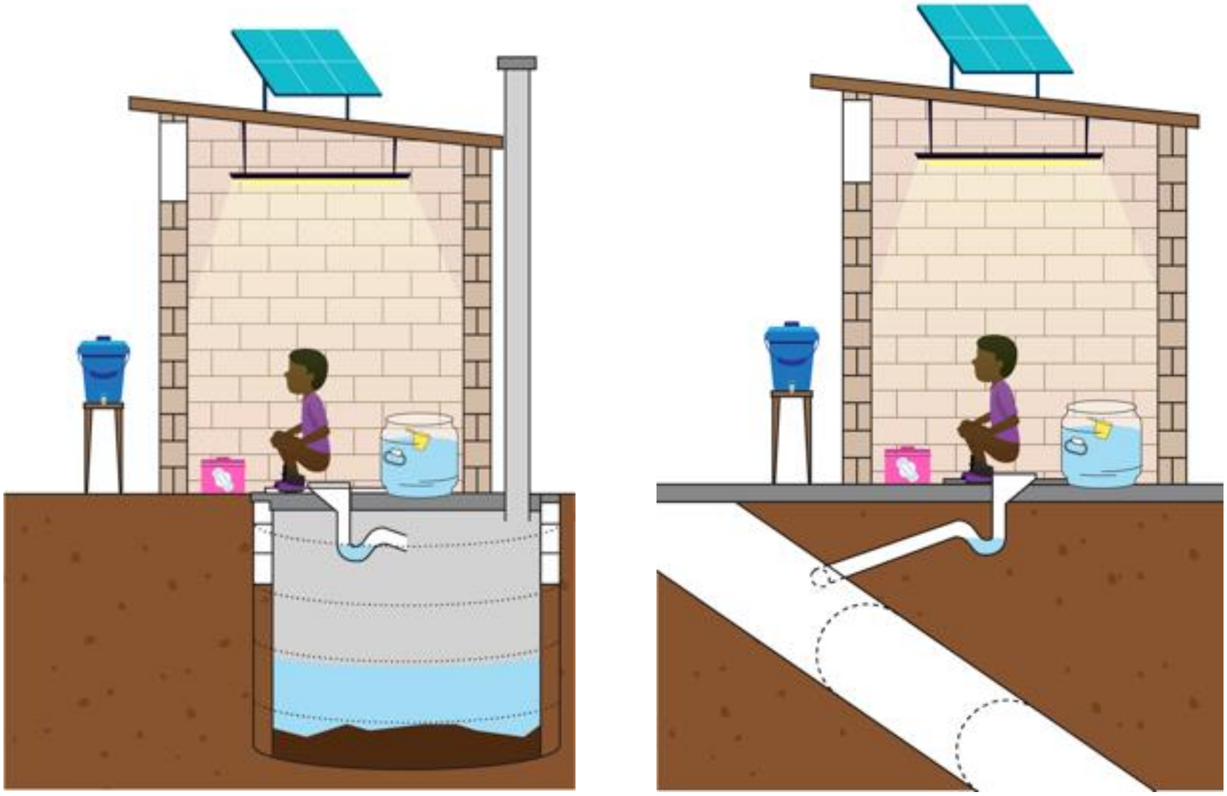

(a) (b)

Figure S2: Graphics of sanitation solutions: (a) pour-flush squatting latrine connected to a lined pit and (b) pour-flush squatting latrine connected to sewer. We used these graphics to explain these sanitation facilities to study participants with the script provided as supplementary text.

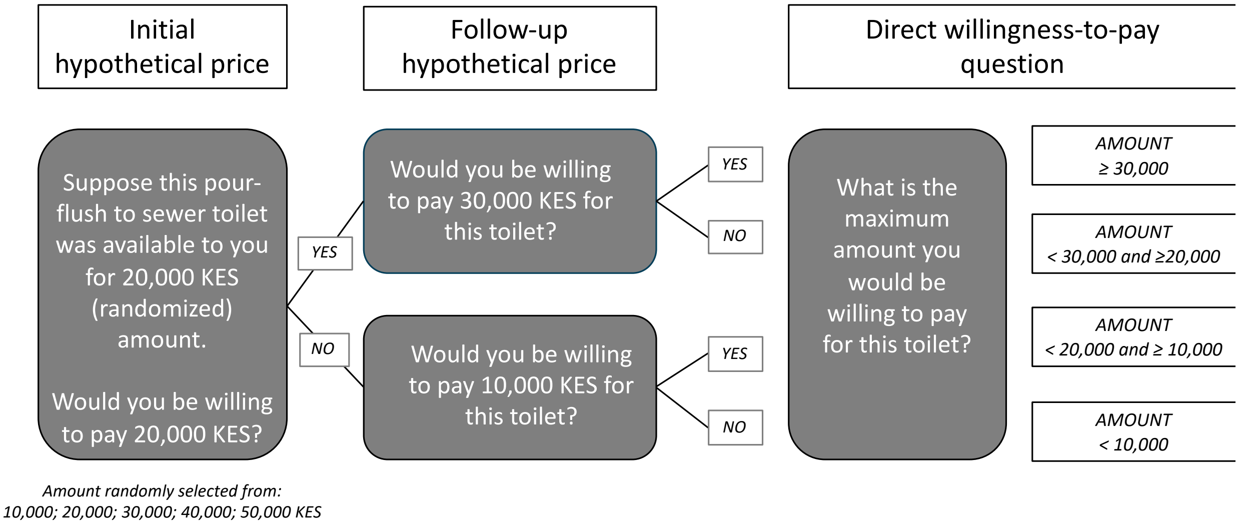

Figure S3: Double-bound dichotomous choice for stated willingness-to-pay.

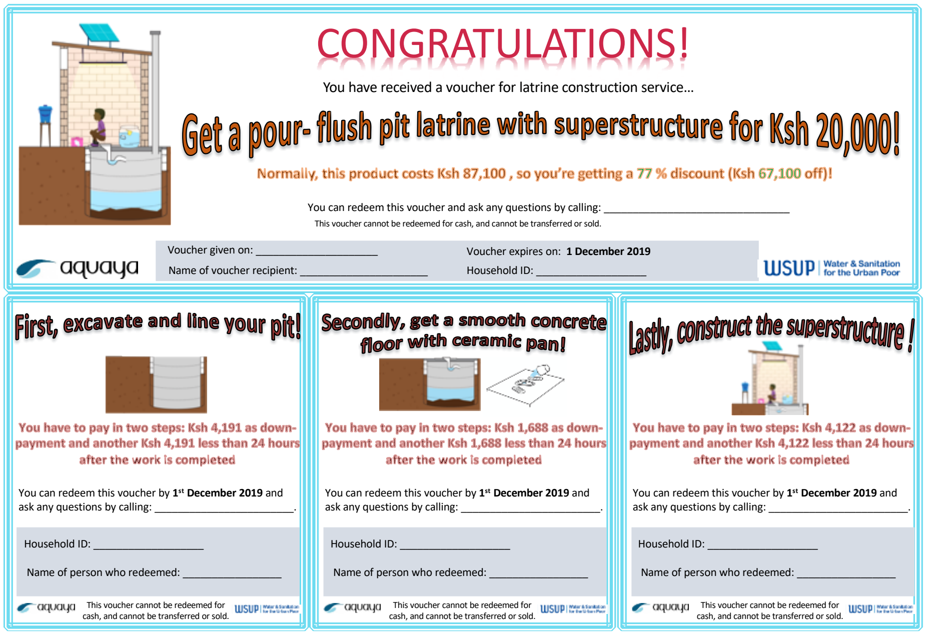

Figure S4: Example voucher for a pour-flush pit latrine.

Table S1: Voucher payments for pour-flush to pit latrine, in KES. The total costs were 87,100 KES (871 USD) for the complete pour-flush latrine including pit, slab, and superstructure. For each stage, the total costs were 36,500 KES (365 USD) for the lined pit excavation, 14,700 KES (147 USD) for the slab, and 35,900 KES (359 USD) for the superstructure.

				Stage 1: Pit excavation			Stage 2: Slab			Stage 3: Superstructure		
**Voucher amount**	**Total cost**	**Discount %**	**Discount**	**Down payment**	**Post-payment**	**Total payment**	**Down payment**	**Post-payment**	**Total payment**	**Down payment**	**Post-payment**	**Total payment**
50,000	87,100	43%	37,100	10,476	10,476	20,953	4,219	4,219	8,439	10,304	10,304	20,608
40,000	87,100	54%	47,100	8,381	8,381	16,762	3,375	3,375	6,751	8,243	8,243	16,487
30,000	87,100	66%	57,100	6,286	6,286	12,572	2,532	2,532	5,063	6,183	6,183	12,365
20,000	87,100	77%	67,100	4,191	4,191	8,381	1,688	1,688	3,375	4,122	4,122	8,243
10,000	87,100	89%	77,100	2,095	2,095	4,191	844	844	1,688	2,061	2,061	4,122

Table S2: Household preferences for latrine types and payment types

	**Landlords/ homeowners (n= 386)**	**Tenants (n= 83)**
**Latrine type**		
Pour-flush to lined pit	70%	49%
Pour-flush to sewer	27%	49%
Container-based sanitation	3%	0%
None	-	1%
**Payment type**		
All upfront (one-time payment)	3%	NA
Installments	90%	NA
Combination	5%	NA
Don’t know	2%	NA

Table S3. Households that redeemed vouchers, all with complete latrine construction.

	Number of households (n= 7)
Paid for latrine from own savings [1]	6
Paid for additional expenses [2]	2
**When redeemed voucher**	
Within past 1 month	3
Within past 3 months	2
Over 3 months ago	1
Don't know	1
**Reasons why household did not redeem voucher earlier** [3]	
Did not have the money	4
Had not discussed with family members	2
Not sure how to redeem	1
Waiting for someone to follow-up	1
Thought it was fraudulent	1
**Satisfaction with voucher redemption and latrine construction process**	
Very satisfied	4
Satisfied	2
Very unsatisfied	1
**Liked about the process**	
Reasonable price	2
Instructions to redeem were clear	4
Masons were professional and pleasant	2
Latrine met my expectations	3
Work was of good quality	3
Other [4]	4
**Disliked about the process**	
Construction was delayed / took a long time	2
Open pit is risky for small children	1

[1] The remaining household borrowed money from neighbors / tenants

[2] One household paid for levelling soil that cost 500 KES (5 USD) and one household paid for a larger pit, amount unknown

[3] Only asked for households that redeemed within the past three months, some households listed multiple responses

[4] Other responses were all reported by one household: the payment process was easy, pit was lined, mason started construction immediately, liked the follow-up.
